# Gastrodin Alleviates Cognitive Dysfunction and Depressive-Like Behaviors by Inhibiting ER Stress and NLRP3 Inflammasome Activation in db/db Mice

**DOI:** 10.3390/ijms19123977

**Published:** 2018-12-10

**Authors:** Tianyuan Ye, Xiangbao Meng, Ruiying Wang, Chenyang Zhang, Shuaibing He, Guibo Sun, Xiaobo Sun

**Affiliations:** 1 Beijing Key Laboratory of Innovative Drug Discovery of Traditional Chinese Medicine (Natural Medicine) and Translational Medicine, Institute of Medicinal Plant Development, Peking Union Medical College and Chinese Academy of Medical Sciences, Beijing, China; yetianyuan2013@163.com (T.Y.); mengxiangbao163@163.com (X.M.); wangruiying8866@163.com (R.W.); zhangchenyang0120@126.com (C.Z.); wenyuxuan2530@163.com (S.H.); 2 Key Laboratory of Bioactive Substances and Resource Utilization of Chinese Herbal Medicine, Ministry of Education, Beijing, China; 3 Key Laboratory of Efficacy Evaluation of Chinese Medicine against Glycolipid Metabolic Disorders, State Administration of Traditional Chinese Medicine, Beijing, China

**Keywords:** diabetic encephalopathy, Gastrodin, NLRP3 inflammasome, ER stress

## Abstract

Patients with diabetes mellitus (DM) suffer more risks from diabetic encephalopathy such as cognitive dysfunction and depressive-like behaviors. Numerous studies show that ER (endoplasmic reticulum) stress and inflammation play important roles in the development of diabetic encephalopathy. *Gastrodin (Gas)*, one major component of *Gastrodia elata*, is traditionally used in central nervous system disorders and is believed to possess anti-inflammatory, anti-apoptotic, and other neuroprotective effects. This present study aims to explore the protective effects of Gas on diabetic encephalopathy. Gas was administrated daily (70 and 140 mg/Kg) for 12 weeks. Meanwhile, the fasting blood glucose and body weight of db/db mice were measured every two weeks. After Gas treatment, the Morris water maze (MWM) test and novel object recognition (NOR) test were performed to assess the learning and memory functions of db/db mice, and the forced swim test was performed to evaluate depressive-like behaviors of db/db mice. Additionally, the expression of ER stress and Nucleotide binding and oligomerization domain-like (Nod) receptor family pyrin domain-containing 3 (NLRP3) inflammasome related proteins were evaluated by using Western blot. Our study suggested that Gas attenuated blood glucose levels and dyslipidemia of db/db mice. It has been shown that Gas could improve learning and memory function and depressive-like behaviors of db/db mice. Moreover, Gas inhibited ER stress and NLRP3 inflammasome activation in the hippocampus. Taken together, this study demonstrates that Gas attenuates the diabetic encephalopathy by inhibiting ER stress and NLRP3 inflammasome activation.

## 1. Introduction

Diabetes mellitus (DM) is a chronic and common metabolic disease with a worldwide prevalence and is associated with high glucose and a deficit in insulin production or sensitivity [1]. Diabetic encephalopathy is one of the main complications of DM [2], it is characterized by atrophy of the brain (gray matter and hippocampus), changes in cerebrovascular morphology and function, impairment of synaptic plasticity, and dysfunction of neuroglia [3]. Patients with DM show neuropsychiatric disorders, and emerging epidemical studies indicate that DM has a close relationship with depression. DM is psychologically demanding given the large and chronic burden placed on patients for the self-management of their disease. Patients have to face some challenges such as adherence to drug treatment, modifications of lifestyle, concerns for complications and disabilities, and psychosocial difficulties at personal and interpersonal levels, leading to depression of DM patients [4]. Moreover, DM patients suffered from depression is often accompanied by cognitive dysfunction [5]. A growing body of evidence identifies DM is an independent risk factor for cognitive dysfunction and dementia such as Alzheimer’s disease (AD) and vascular dementia, 50% of DM patients will develop cognitive impairments as they age [3,6]. Oxidative stress, ER stress, inflammation [7], neurotrophic impairment, neuron death, insulin resistance [8], and increased advanced glycation end products are related to diabetic encephalopathy [9]. 

High glucose initiates and facilitates inflammation in the course of diabetic encephalopathy progression. Nucleotide binding and oligomerization domain-like (Nod) receptor family pyrin domain-containing 3 (NLRP3) inflammasome is a cytosolic sensor of exogenous pathogens and endogenous damage-associated molecular patterns (DAMPs) [10]. Once NLRP3 inflammasome is activated, NLRP3 will assemble with cysteine protease caspase-1 and adaptor protein ASC (apoptosis-associated speck-like protein containing a card), leading to the cleavage and activation of caspase-1. Caspase-1 catalyzes the transformation of precursors IL-1β (interleutin-1β) and IL-18 (interleutin-18) into IL-1β and IL-18. The production and release of IL-1β and IL-18 induces various forms of inflammatory reaction, thus contributing to the pathogenesis and progression of CNS diseases [11,12,13]. MPTP-driven NLRP3 inflammasome activation in microglia plays a central role in dopaminergic neurodegeneration [14], and the activation of NLRP3 inflammasome can mediate fatigue-like behaviors in mice via neuroinflammation [15]. Our previous study indicated NLRP3 was activated in the hippocampus of db/db mice [16]. Thus, NLRP3 can influence the development of neuron death, inflammation, and may have an impact on diabetic encephalopathy.

The accumulation of unfolded and misfolded proteins in the endoplasmic reticulum (ER) caused by multiple disturbances will trigger the ER stress response [17]. ER stress contains three signaling pathways: PERK (protein kinase R like endoplasmic reticulum kinase), IRE (inositol-requiring enzyme), and ATF6 (activating transcription factor 6), which are activated by the ER chaperone GRP78 (glucose regulated protein 78). There is growing evidence that ER stress is involved in the pathogenesis of metabolic pathologies and the number of diseases involving in ER stress has also increased [18]. In vivo and in vitro studies show that ER stress is associated with high-glucose induced neuron damage and diabetic encephalopathy [19]. It has been reported that ER stress is related to NLRP3 inflammasome activation in liver failure and inhibition of ER stress can ameliorate liver injury and hepatocyte death [17]. As one of the inflammasome family members, NLRP3 can be activated by PAMPs (pathogen-associated molecular patterns), extracellular ATP (adenosine triphosphate), ROS (reactive oxygen species), accumulation of Aβ (amyloid-β), aggregated and misfolded proteins and crystalline substances [20,21], suggestive of its critical role in inflammatory responses. ER stress can induce the activation of NLRP3 inflammasome in high fat diet-induced obese rats and affect neuronal plasticity-related protein levels [22]. However, the precise link between ER stress and NLRP3 in diabetic encephalopathy is still unknown.

**Figure 1:**
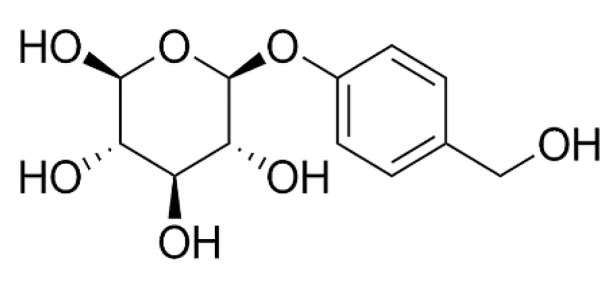
Chemical structure of Gas; molecular weight is 286.3; molecular formula is C_13_H_18_O_7_.

Due to the complexity of diabetic encephalopathy, increasing interests have switched to the natural components from herbs. Gastrodin (Gas, its molecular weight is 286.28, and its molecular structure is shown in Figure 1) is one of the main bioactive components derived from the rhizome of *Gastrodia elata*, a kind of traditional Chinese medicine used for dementia, stroke, dizziness, headache, cardiovascular diseases and other diseases [23,24]. Gas can pass the blood–brain barrier (BBB) and is found in several rat brain regions. In the last few years, researchers focused much attention on the effects of Gas in central nervous systems [25], and it’s now demonstrated that Gas exhibits its neuroprotective effects through inhibition of apoptosis, oxidative stress, and the release of inflammatory cytokines such as IL-1β, TNF-α (tumor necrosis factor-α) [26,27]. Gas attenuates seizures by modulating mitogen-activated protein kinase-associated inflammatory responses in mice [28]. It ameliorates subacute phase cerebral ischemia-reperfusion injury by inhibiting inflammation and apoptosis in rats [29]. In addition, Gas increased the expression of BDNF (brain derived neurotropic factor) and reversed the traumatic stress-induced depressed-like symptoms in rats [30]. Even though there are many researches clarifying the protective effects of Gas on several brain disease models, no one has focused on its effects on diabetic encephalopathy. 

In view of the lack of an understanding about Gas in diabetic encephalopathy, this present study investigated inhibitory roles of Gas on ER stress and NLRP3 inflammasome activation. We also demonstrated that Gas could improve hyperglycemia, dyslipidemia, and insulin resistance in db/db mice. Moreover, it could ameliorate cognitive dysfunction and depression-like behaviors of db/db mice through the inhibition of ER stress and NLRP3 inflammasome activation. 

## 2. Results

### 2.1. Effects of Gas on Body Weight and Fasting Blood Glucose (FBG) on db/db Mice

**Figure 2:**
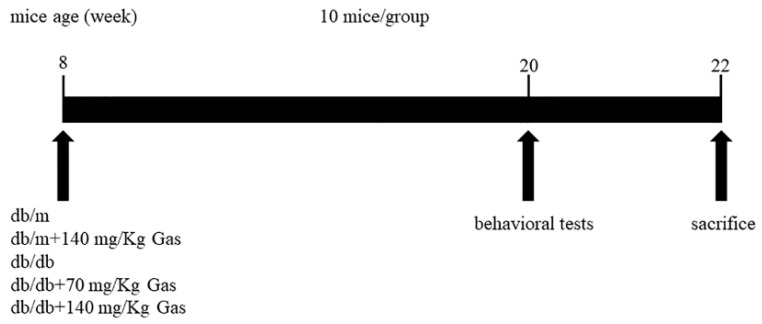
Summary of the experimental design.

**Figure 3:**
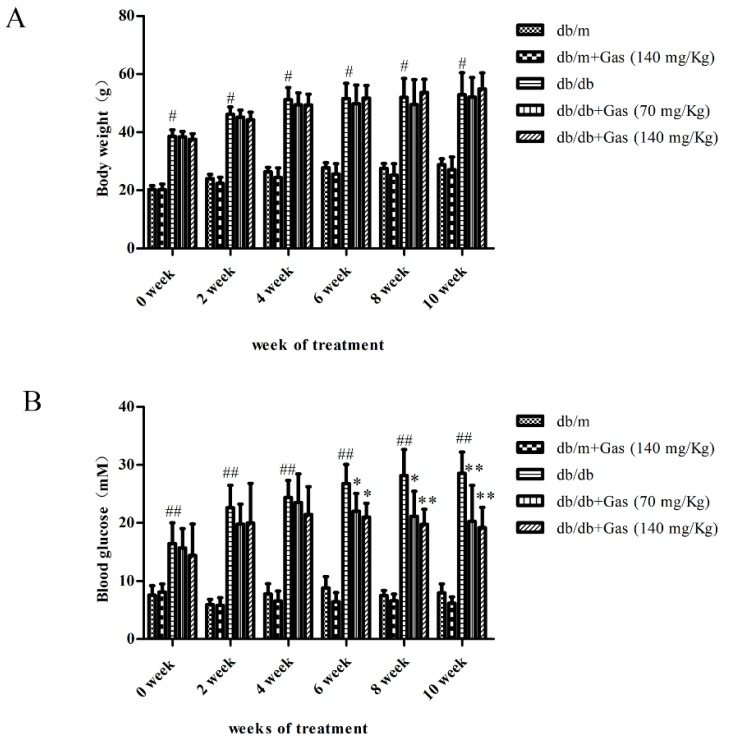
Effects of Gastrodin (Gas) on body weight and fasting blood glucose of db/db mice. (**A**) Body weight of mice in each group during 12 weeks of treatment. (**B**) Fasted blood glucose level of mice in each group during 12 weeks of treatment. # *p* < 0.05, ## *p* < 0.01, compared with the db/m group. * *p* < 0.05, ** *p* < 0.01, compared with the db/db group.

The experimental design was shown in Figure 2. The db/db mice were spontaneous diabetes model accompanied with high blood glucose and fat. The daily administration of Gas (70 and 140 mg/Kg) was carried out for 12 weeks. To determine the effects of Gas on body weight and fasting blood glucose of db/db mice, we monitored these two indicators every two weeks. As shown in Figure 3A,B, the body weight and FBG of db/db mice were higher than non-diabetic db/m mice (*p* < 0.01). Gas has no influence on the body weight of db/db mice during the experiment. The hypoglycemic effect of Gas was evident after treatment with Gas for 6 weeks, FBG was reduced significantly in a dose-dependent manner (*p* < 0.05, *p* < 0.01). These results indicated the effective and sustained hypoglycemic effects of Gas in diabetic mice.

### 2.2. Gas Improved Dyslipidemia and Insulin Resistance in db/db Mice

**Figure 4:**
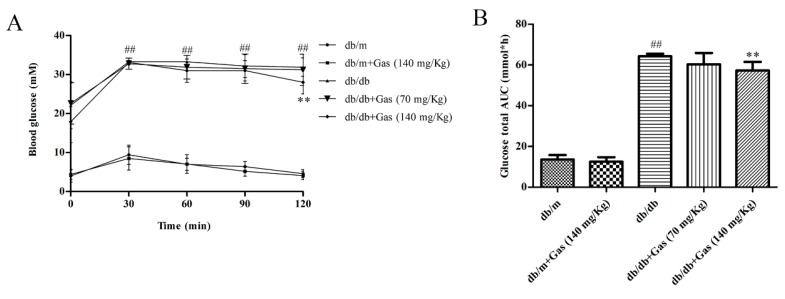
Effects of Gas on insulin resistance of db/db mice. (**A**) Curve of blood glucose level in OGTT. (**B**) Glucose total AUC in OGTT. ## *p* < 0.01, compared with the db/m group. ** *p* < 0.01, compared with the db/db group.

OGTT (oral glucose tolerance test) was conducted to evaluate the effect of Gas on glucose tolerance of db/db mice. As shown in Figure 4A,B, blood glucose level and glucose total area under the curve (AUC) in db/db mice was significantly higher than db/m mice (*p* < 0.01). However, 140 mg/Kg Gas treatment could decrease blood glucose level after administration of glucose for 120 min (*p* < 0.01). In addition, high doses of Gas could also decrease AUC (*p* < 0.01). These results indicated the protective effects of Gas on glucose tolerance.

**Figure 5:**
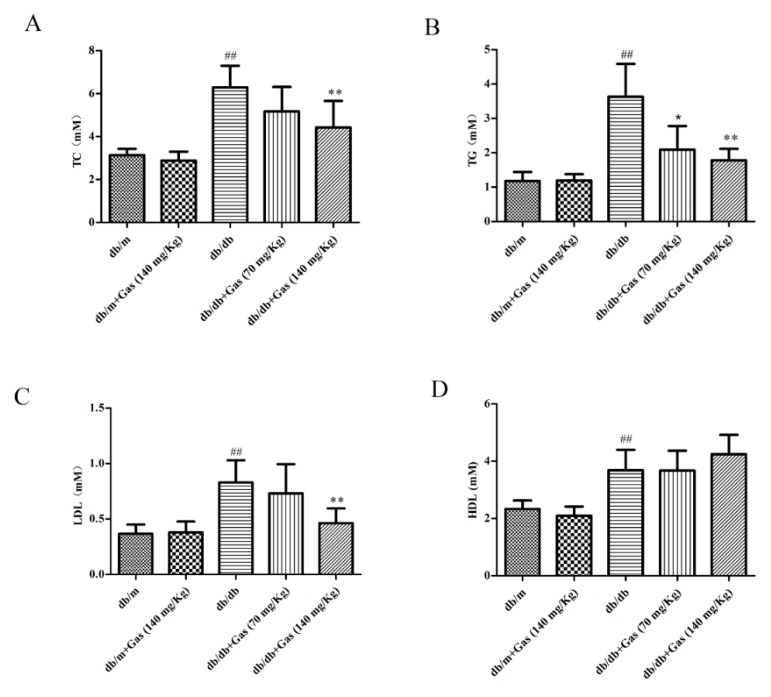
Gas improves dyslipidemia in db/db mice. (**A**) Levels of total cholesterol (TC) in plasma samples of mice after 12 weeks of treatment. (**B**) Levels of triglyceride (TG) in plasma samples of mice after 12 weeks of treatment. (**C**) Levels of low-density lipoprotein cholesterol (LDL-C) in plasma samples of mice after 12 weeks of treatment. (**D**) Levels of high-density lipoprotein cholesterol (HDL-C) in plasma samples of mice after 12 weeks of treatment. Values are represented as means ± SD for 10 mice in each group. ## *p* < 0.01, compared with the db/m group. * *p* < 0.05, ** *p* < 0.01, compared with the db/db group.

Diabetes mellitus is characterized by dyslipidemia. As the results shown in Figure 5, the contents of total cholesterol (TC), triglyceride (TG), high-density lipoprotein cholesterol (HDL-C), and low-density lipoprotein cholesterol (LDL-C) were increased significantly in db/db mice compared with the normal group (*p* < 0.01). Treatment with 140 mg/Kg Gas for 12 weeks could significantly decrease the levels of these three indicators (*p* < 0.01). However, although 140 mg/Kg Gas could increase the amount of HDL-C, no significant change was found in the result. Thus, our results demonstrated that Gas could improve dyslipidemia in db/db mice.

### 2.3. Gas Improved Learning and MEMORY Abilities of db/db Mice

**Figure 6:**
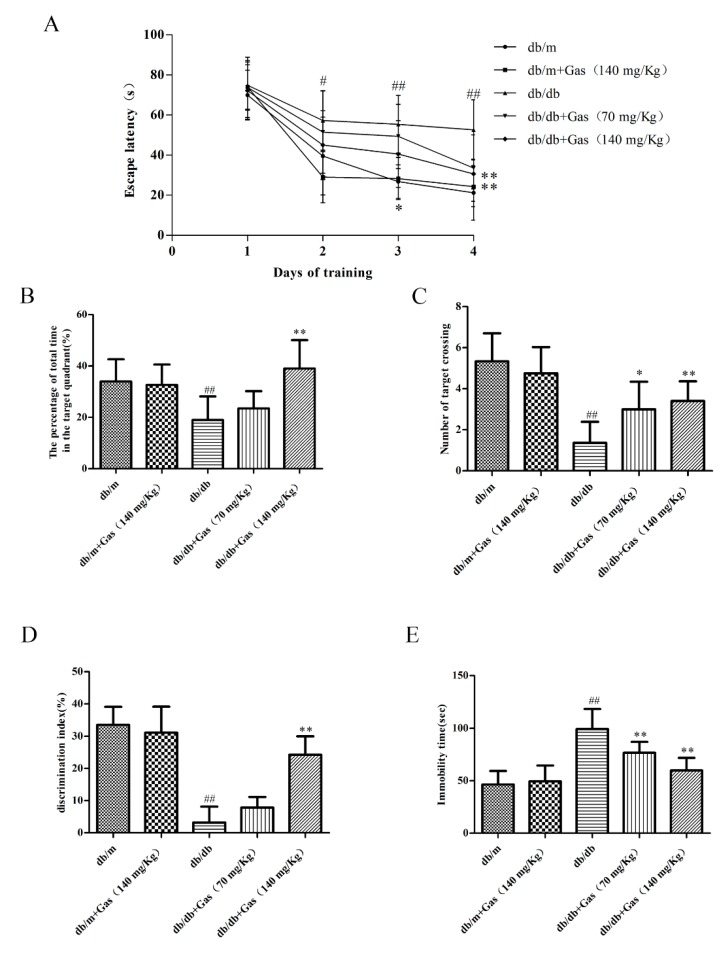
Gas improves cognitive dysfunction and depression-like behaviors in db/db mice. (**A**) Escape latency of the four-day hidden-platform test. (**B**) Number of target crossing in the probe trial. (**C**) Percentage of total time spent in target quadrant in the probe trial. (**D**) Discrimination index of the db/db mice. (**E**) Immobility time of the db/db mice. All data are expressed as means ± SD for 10 mice in each group. # *p* < 0.05, ## *p* < 0.01, compared with the db/m group. * *p* < 0.05, ** *p* < 0.01, compared with the db/db group.

The learning and memory abilities of db/db mice were measured by Morris water maze (MWM) and novel object recognition (NOR) test. In MWM test, spatial learning function was measured by the escape latency during the four-day training, which declined progressively during training days, as shown in Figure 6A. On the first day, every group spent approximately same time to get to the platform. Since the second day, the escape latency of the db/db groups was notably longer than the db/m group (*p* < 0.01), indicating memory deficits in db/db mice. On the fourth day, Gas treated mice spent less time than the db/db mice finding the hidden platform (*p* < 0.01). Probe test was conducted on the fifth day with the platform removed, the percentage time spending in the target quadrant and the number of platform crossings were characterized as learning and memory ability. These two indicators were significantly increased in db/db group when compared with db/m group (*p* < 0.01). However, Gas could reduce the percentage time spending in the target quadrant and the number of platform crossings (*p* < 0.05, *p* < 0.01), as shown in Figure 6B,C. 

The discrimination index (DI) in NOR test could reflect recognition memory of mice. As the results shown in Figure 6D, DI was decreased dramatically (*p* < 0.01), indicating memory deficits of db/db mice. 140 mg/Kg Gas could increase the DI, indicating that Gas could improve cognitive deficits in db/db mice. These results reflected protective effects of Gas on learning and memory abilities of db/db mice.

### 2.4. Gas Ameliorates Depression-Like Behaviors in db/db Mice

The forced swim test was conducted to evaluate depression-like behaviors of db/db mice. In Figure 6E, immobility time was increased significantly in db/db mice than the db/m mice (*p* < 0.01), which was considered more depressive than normal group. Gas treatment (70 mg/Kg and 140 mg/Kg) could obviously decrease the immobility time of db/db mice (*p* < 0.01). These results suggested that Gas could improve depression-like behaviors in db/db mice.

### 2.5. HE (Hematoxylin and Eosin) and NISSL Staining

**Figure 7:**
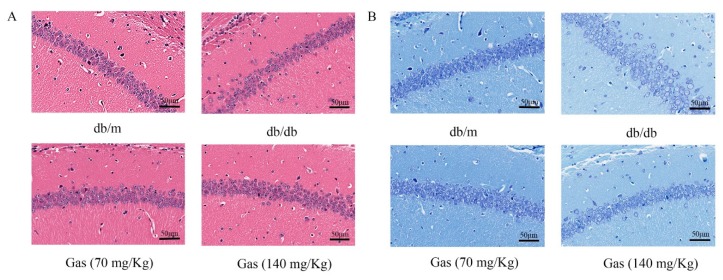
Gas prevented neuronal apoptosis in the hippocampus of db/db mice. (**A**) HE staining in the hippocampal CA1 region for each group. (**B**) Nissl staining in the hippocampal CA1 region for each group.

HE staining was performed to evaluate protective effects of Gas on neuronal loss and damage in the hippocampus of db/db mice. As shown in Figure 7A, intact neurons could be obviously observed in HE staining of db/m group. In db/db mice, nuclei pyknosis and damaged neurons presented in the CA1 region of hippocampus. After Gas treatment (70 or 140 mg/Kg), nuclei pyknosis were reduced in this region of hippocampus in db/db mice. The Nissl staining of the hippocampal CA1 area in brain sections was utilized to examine whether neurons were damaged in db/db mice. In Figure 7B, many pyknotic neurons were scattered disorderly in the hippocampal CA1 region of db/db mice. Neurons also showed light staining, indicating that cells were damaged, as multiple Nissl bodies disappeared in neurons. However, in the Gas group, dark staining of neurons could be observed in the CA1 region of hippocampus, and neurons were arranged orderly in this region. Thus, Gas can improve hippocampal damage in db/db mice.

### 2.6. Gas Inhibits ER Stress and NLRP3 Inflammasome Activation of db/db Mice

**Figure 8:**
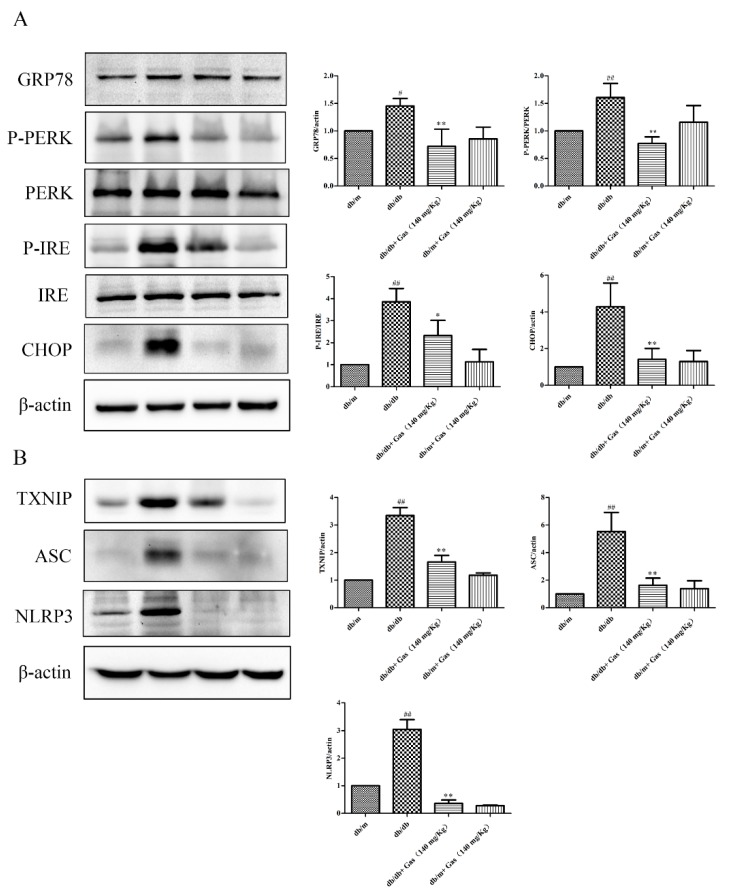
Gas inhibits ER stress in the hippocampus of db/db mice. (**A**) Representative protein bands and Western blot analysis of GRP78, P-PERK, PERK, IRE, P-IRE, CHOP in the hippocampus of each group. (**B**) Representative protein bands and Western blot analysis of TXNIP, NLRP3, ASC in the hippocampus of each group. Values are represented as means ± SD for three mice in each group. ^#^ *p* < 0.05, ^##^ *p* < 0.01, compared with the db/m group. * *p* < 0.05, ** *p* < 0.01, compared with the db/db group.

To explore the important roles of Gas on diabetic encephalopathy, we used Western blot to measure expression of related proteins. As the results shown in Figure 8A, ER stress was induced under hyperglycemia and dyslipidemia conditions, as the expression of GRP78, CHOP (CCAAT/enhancer binding protein homologous protein) and the ratio of P-PERK/PERK, P-IRE/IRE were significantly increased in the hippocampus of db/db mice than the db/m group (*p* < 0.05, *p* < 0.01). Gas treatment in a dose of 140 mg/Kg could dramatically decrease their expression (*p* < 0.01). NLRP3 inflammasome activation indicate the initiation of inflammatory responses. TXNIP (thioredoxin-interacting protein) can interact with NLRP3 and induce the activation of NLRP3 inflammasome. As the results shown in Figure 8B, the expression of TXNIP, NLRP3, and ASC was increased significantly (*p* < 0.01). Gas in a dose of 140 mg/Kg has a significant effect on the activation of NLRP3 inflammasome (*p* < 0.01). 

## 3. Discussion

To our knowledge, this is the first study to clarify neuroprotective effects of Gas in db/db mice. In this study, diabetic mice showed cognitive dysfunction and depression-like behaviors, accompanied with hyperglycemia, dyslipidemia, and central nervous system inflammation. Treatment with Gas for 12 weeks could ameliorate cognitive deficits, depression-like behaviors, and insulin resistance in db/db mice. Previous DM studies on humans, mice, and rats showed dyslipidemia and hyperglycemia [16,31], consistent with these findings in the present study. Our results showed that Gas could decrease fasting blood glucose and improve dyslipidemia of diabetic mice, as the levels of TC, TG, and LDL-C were significantly reduced after administration with Gas for 12 weeks. However, Gas has no significant influence on the level of HDL-C, indicating its target is not on the production of HDL-C. Moreover, HE and Nissl staining reflected the effects of Gas on hippocampal neurons in db/db mice, the results suggested that Gas could modify morphology of neurons in db/db mice. We also found inflammation and ER stress were associated with diabetic encephalopathy, as ER stress, TXNIP, and NLRP3 inflammasome is activated in the hippocampus, but Gas treatment could reverse these changes. Our study investigated the inhibitory roles of Gas in ER stress and NLRP3 inflammasome activation in response to diabetes, indicating its neuroprotective effects on diabetic encephalopathy. 

The prevalence of DM has reached epidemic levels globally, leading to enormous human, economic, and social cost worldwide [32]. Now there are more than 400 million people suffering from diabetes, and the number is predicted increased to 642 million by 2040. As patients must be faced with longtime drug administration, self-nursing, food restriction, and diabetic complications during the progression of diabetes, patients are more likely to experience depressive symptoms [33]. Worldwide, about half of diabetic patients have severe depression [34]. Also, in present study, we investigated that db/db mice exhibited depression and Gas treatment could improve this kind of symptom. In addition, cognitive impairment is another serious complication and it aggravates with ages, it is related to a decrement in work productivity and overall lower quality of life [35]. The MWM test and NOR test indicated that db/db mice suffered from cognitive dysfunction, learning and memory function was impaired during the DM progression. Gas treatment could improve the cognitive function of diabetic mice. Key causative pathways in diabetes-associated cognitive dysfunction and depression need to be identified in order to develop course-modifying therapies.

Gas, the main bioactive component in the rhizome of *Gastrodia elata*, has shown the properties of hypoglycaemic, anti-inflammatory, and anti-apoptotic properties. Traditionally, Gas is used on central nervous system disorders such as dementia [36], Alzheimer’s disease [37], Parkinson’s disease [38], affective disorders, and so on. New studies find that Gas can protect against alcohol-induced liver injury in mice (50, 80, 100mg/Kg) and rats (100 mg/Kg) through inhibition of oxidative stress [39,40]. Gas can also inhibit high-glucose-induced human retinal endothelial cell apoptosis by regulating the SIRT1/TLR4/NF-κBp65 signaling pathway [41]. Taking these results together, we hypothesized that Gas can improve diabetic encephalopathy. We used two doses in the experiment (70 mg/Kg and 140mg/Kg) to identify our thoughts. Our results suggest that Gas can ameliorate blood glucose and dyslipidemia of db/db mice. Gas can also inhibit ER stress and NLRP3 inflammation activation, improving cognitive dysfunction and depressive-like behaviors.

Inflammation plays a critical role during the onset and progression of diabetes encephalopathy. NLRP3 inflammasome consists of three components: the sensor nucleotide oligomerization domain (NOD)-like receptors (NLRs), the adaptor protein ASC, and the effector molecule caspase-1 [42]. NLRP3 can be activated by two steps: the expression of inflammasome components and their functional activation [17]. Ion fluxes, ROS, and phagosome destabilization are main factors that lead to the activation of NLRP3 inflammasome. Now researchers find that NLRP3 inflammasome can be activated under high glucose conditions [43,44]. The levels of CCL2 (chemokine ligand 2), IL-1β, and other proinflammatory cytokines are significantly reduced in NLRP3-deficient mice [45]. Inhibiting the activation of NLRP3 inflammasome with MCC950 can promote non-phlogistic clearance of Aβ and cognitive function in APP/PS1 mice [46], it can also ameliorate cognitive dysfunction and depressive-like behaviors in db/db mice [16]. NLRP 3 inflammasome is also activated in other diabetic complications such as diabetic nephropathy [47], diabetic cardiomyopathy [44], diabetic retinopathy, and diabetic vascular endothelial dysfunction [48]. Consistent with these findings, in our study, we found the expression of NLRP3 and ASC in the hippocampus of db/db mice is significantly increased, Gas could notably decrease the activation of NLRP3 inflammasome.

Although these data show a close relationship between diabetic encephalopathy and NLRP3 inflammasome activation, the mechanisms by which it is activated is still unclear. Lipid environment induces ER stress, TXNIP expression, and inflammation [49]. ER stress in hippocampus was presented in db/db mice suffering from diabetic encephalopathy [19]. Similarly, in our study, we investigated ER stress induced by hyperglycemia and dyslipidemia, as the expression of GRP78, CHOP, and the ratio of P-PERK/PERK, P-IRE/IRE were dramatically increased in the hippocampus of db/db mice. NLRP3 expression is enhanced by PERK overexpression and the production of IL-1β could be decreased through *Perk* knockdown. A new study indicates that CHOP can interact with the *NLRP3* gene, and it is a potential transcriptional factor for the NLRP3 expression downstream of PERK [17]. In addition, TXNIP can interact with many proteins including NLRP3 [50]. Studies confirmed that TXNIP plays an important role in the NLRP3 inflammasome activation and ER Stress-induced cell death [51]. Our results demonstrate that TXNIP is dramatically increased in db/db mice, the overexpression of NLRP3 and its binding partner TXNIP might contribute to ER stress-induced neuroinflammation.

**Figure 9:**
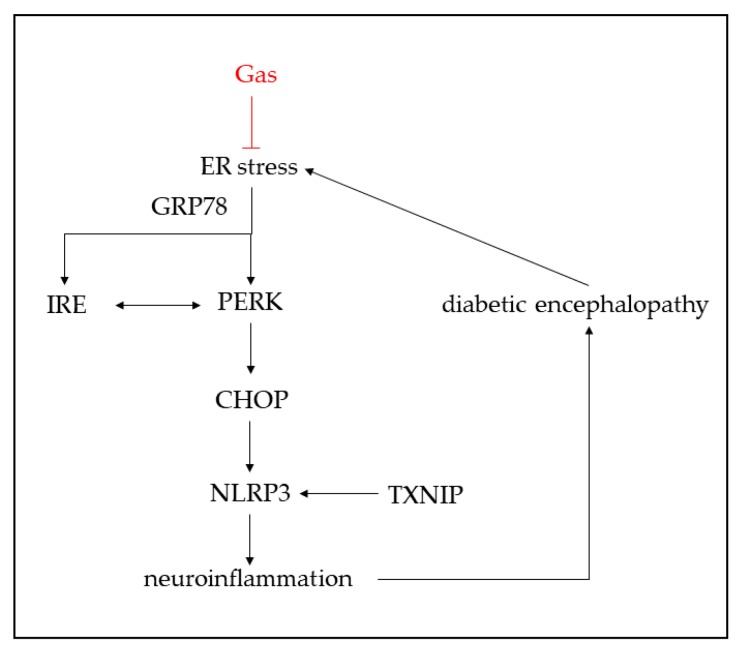
Schematic representation of Gas-mediated neuroprotective effect in db/db mice. Gas inhibited ER stress and NLRP3 inflammasome activation.

In summary, our present findings demonstrate that Gas can improve hyperglycemia and dyslipidemia in db/db mice, the morphology of neurons can also be improved by Gas. Moreover, Gas ameliorates cognitive dysfunction and depression-like behaviors of db/db mice by inhibiting ER stress and NLRP3 inflammasome activation (Figure 9). These results elucidate that Gas exhibits the neuroprotective effects in DM. If these effects of Gas are validated in clinical trials, it might be a promising avenue for diabetic encephalopathy therapy.

## 4. Materials and Methods

### 4.1. Reagents and Materials

Gastrodin (CAS NO: 62499-27-8, purity > 99%) was purchased from Shanghai Winherb Medical Science Company (Shanghai, China). The kits for detecting TG, TC, LDL-C, and HDL-C were purchased from Biosino Biotechnology & Science Inc. (Beijing, China). Primary antibodies against NLRP3, TXNIP, IRE, P-IRE were from Abcam (Cambridge, MA, USA). Primary antibodies against CHOP, PERK, P-PERK, ASC were provided by Santa Cruz Biotechnology (Santa Cruz, CA, USA).

### 4.2. Animals

Male 7-week old diabetic mice with a homozygous mutation of the leptin receptor (C57BLKS/J-lepr^db^/lepr^db^) and age-matched non-diabetic mice (db/m) were purchased from the Model Animal Research Center of Nanjing University (Nanjing, China). The mice were housed in a room with 12 h light/dark cycle at 22–24 °C and allowed free access to food and water. All laboratory procedures were performed according to the Research Ethics Committee of the Chinese Academy of Medical Sciences and Peking Union Medical College, Beijing, China (SCXK 2014-0001). After 1 week’s adaptation, the diabetic db/db mice were randomly divided into three groups (*n* = 10): model group, Gas (70 mg/Kg) group, Gas (140 mg/Kg) group. The db/m mice were divided into two groups (*n* = 10): the control group and Gas (140 mg/Kg) group. All animals were orally gavaged with Gas or saline daily for 12 weeks. Body weight and fasting blood glucose level were measured every 2 weeks.

### 4.3. Oral glucose Tolerance Test (OGTT)

OGTT was performed as previous methods with tiny modifications [31]. OGTT was conducted after an overnight fasting. Mice were orally gavaged with glucose solution (1g/Kg), followed by blood glucose measurement at 0, 30, 60, 90, and 120 min. Blood glucose was detected by an automatic glucometer (One Touch Ultra, Lifescan, USA).

### 4.4. Morris Water Maze (MWM)

Spatial learning and memory were measured by using the MWM test. The MWM test was performed according to our previous method with minor modifications [16]. Briefly, the circular pool (100 cm in diameter, 50 cm in height) was divided into four quadrants marked with circle, diamond, square, and triangle on the wall of the pool. The pool was filled with water and the temperature was controlled 21–23 °C. A circular black platform (9 cm in diameter) was placed in the center of one quadrant, 1 cm under the water. The test contained 4-day training and 1-day probe test. During training days, mice were trained thrice daily with at least 1 h intervals. In each trail, mice were placed in the water facing the wall at one of the four different starting positions and found the platform. The time mice spent on finding the hidden platform was recorded as escape latency. The trail stopped until the mice found the platform. If the mice were unable to find the target within 90 s, the escape latency was recorded as 90 s. Whether or not the mice could find the platform, they were allowed to stay on it for 15 s. The probe test was performed on the fifth day to evaluate spatial learning and memory of the diabetic mice. The platform was removed, the mice were placed on the opposite position of the platform and allowed to swim for 90 s. The number of mice crossing the previous platform position and time spent in the target quadrant were recorded. The test was performed from 8:00 a.m. to 5:00 p.m. under dim illuminations.

### 4.5. Novel Object Recognition (NOR)

The NOR test was conducted to evaluate memory function, which based on the natural preference of rodents for novel objects. The experimental cage (38 cm × 38 cm × 38 cm) was located in a quiet and dim testing room. A video camera was located above the cage and connected to a computer to capture data. Mice were first habituated the environment for 2 days (10 min/day), they were allowed to explore the empty open field freely. The two-day training test was conducted 24 h after 2 days of habituation. Two identical objects were placed in the back left and right corners of the cage, 8 cm from the walls. Mice were placed in the cage facing the wall and opposite to the objects, allowed to explore the objects freely for 5 min. If the mice did not explore the objects or have preference for one object than the other on this training day, they were excluded from analysis. During the test, one of the familiar objects was replaced with a novel object and mice were again allowed to explore for 5 min. All subjects should present similar texture, size and colors, but have different shapes. Object exploration was defined as the mice sniffing the object with nose or touching the object at a distance of no more than 2 cm. Sitting on the object was not considered as exploration. Mice would spend more time exploring novel subject than the familiar one because of its instinct for novelty. Between each individual test, the field and objects were thoroughly wiped with 75 % ethanol. The lack of preference for novel object indicating a decrease of recognition memory. Discrimination index (DI) was used to evaluate the recognitive ability of mice. DI (%) = (TN – TF)/(TN + TF) × 100% (TN = the time spent on the novel object; TF = the time spent on the familiar object). 

### 4.6. Forced Swim Test

Forced swim test was performed to assess the effects of Gas on depression-like behavior of db/db mice. The mice were placed in a cylinder (13 cm in diameter, 20 cm in height) that containing 25 °C water 16 cm deep. The test was lasted for 6 min with a camera above the cylinder. The immobility time was measured for the last 4 min. Immobility was scored when the mouse remained floating passively in water without active movements of forepaws.

### 4.7. Measurement of Plasma Lipids

After 12 weeks of administration, blood samples were collected after an overnight fasting for 12 h and were centrifuged at 3500 rpm for 15 min. Plasma TC, TG, LDL-C, HDL-C levels were measured by Hitachi 7600 Automatic Biochemistry Analyzer (Tokyo, Japan).

### 4.8. HE and Nissl Staining

Excised brain specimens were immersed in 4 % paraformaldehyde for 24 h, embedded in paraffin, and then cut into tissue sections (4–6 μm thick). For HE and Nissl staining, we conducted the experiments according to our previously methods [16]. Light microscopy was used to observe the images (Life Technologies, Leica, Vista, CA, USA).

### 4.9. Western Blot Analysis

The hippocampal tissues were homogenized in ice-cold RIPA (radio immunoprecipitation assay) buffer supplemented with protease inhibitors. Protein concentration was measured by bicinchoninic acid (BCA) method. Proteins were separated with 8–12% SDS-PAGE and then transferred onto nitrocellulose membranes (Bio-Rad, Hercules, CA, USA). The membranes were blocked with 5% non-fat milk blocking buffer for 2 h at room temperature and incubated with the following primary antibodies overnight at 4 °C: GRP78 (1:200), PERK (1:200), P-PERK (1:200), IRE (1:1000), P-IRE (1:1000), CHOP (1:200), TXNIP (1:1000), NLRP3 (1:1000), ASC (1:200), β-actin (1:1000). The membranes were then washed and incubated with proper HRP-conjugated secondary antibodies. Protein bands were visualized after development using an enhanced chemiluminescence solution for 5 min. Image Lab was used to determine relative density of protein bands.

### 4.10. Statistical Analysis

Experimental results in this research were expressed as means ± SD. Differences between groups were determined by one-way analysis of variance (ANOVA) using SPSS 17 (SPSS, Chicago, IL, USA), followed by least significant difference post hoc comparison test. Indexes in MWM tests were analyzed by repeated-measure two-way ANOVA. *p* < 0.05 was regarded as statistically significant.
